# *C*_2_-symmetric bisamidines: Chiral Brønsted bases catalysing the Diels-Alder reaction of anthrones

**DOI:** 10.3762/bjoc.4.28

**Published:** 2008-08-07

**Authors:** Deniz Akalay, Gerd Dürner, Jan W Bats, Michael W Göbel

**Affiliations:** 1 Johann Wolfgang Goethe University Frankfurt, Institute of Organic Chemistry and Chemical Biology, Max-von-Laue-Str. 7, D-60438 Frankfurt am Main, Germany.

**Keywords:** Asymmetric Catalysis, Bisamidines, Brønsted base, Diels-Alder reaction, Organocatalysis

## Abstract

*C*_2_-symmetric bisamidines **8** have been tested as chiral Brønsted bases in the Diels-Alder reaction of anthrones and *N*-substituted maleimides. High yields of cycloadducts and significant asymmetric inductions up to 76% *ee* are accessible. The proposed mechanism involves proton transfer between anthrone and bisamidine, association of the resulting ions and finally a cycloaddition step stereoselectively controlled by the chiral ion pair.

## Introduction

**Scheme 1:**
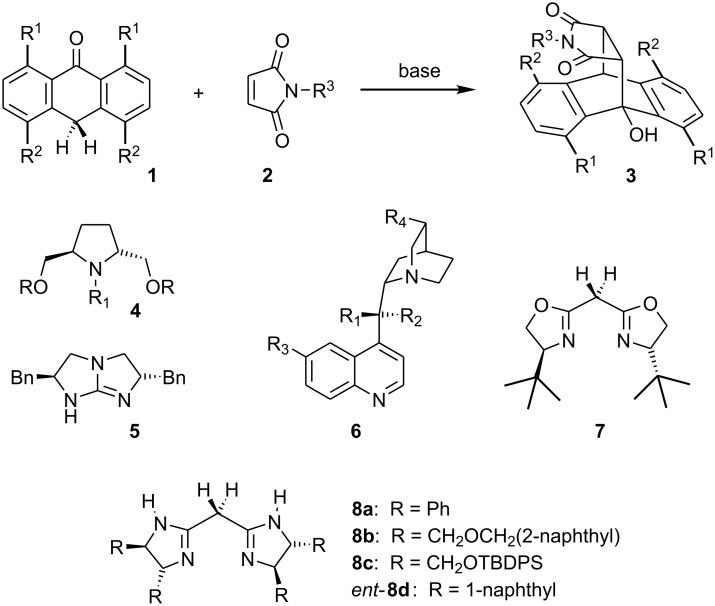
Diels-Alder reaction of anthrones **1** and maleimides **2** catalyzed by chiral Brønsted bases **4**–**8**.

The cycloadditions of anthrones **1** and *N*-substituted maleimides **2** are prominent examples of asymmetric catalysis exerted by chiral Brønsted bases. Moderate to excellent stereoselectivities of products **3** have been reported using pyrrolidines **4** [1–2], cyclic guanidine **5** [3], or cinchona alkaloids **6** [4] as catalysts. Recently, we could promote this type of cycloaddition by metal-free bisoxazolines **7** in up to 70% *ee*, in spite of their limited Brønsted-basicity [5] (Scheme 1).

**Scheme 2:**
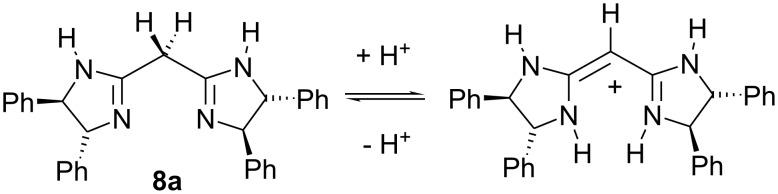
Protonation states and tautomerism of *C*_2_-symmetric bisamidine **8a** [6].

Our study was motivated by the structural similarity of bisoxazolines **7** and bisamidines **8**. Bisamidines **8**, readily accessible from malonodinitrile in two steps, prefer the conjugated tautomeric form (enamine-imine) in the monoprotonated state, which is characterised by an almost planar structure [6] (Scheme 2).

The aqueous p*K*_a_ of **8**·H^+^ is approximately 11, sufficient to allow deprotonation of anthrones **1** (p*K*_a_ around 10, [7–8]) by bisamidines to a significant extent. Here we report on the use of neutral bisamidines **8** as asymmetric Brønsted base catalysts in the cycloaddition of anthrones **1** and maleimides **2**.

## Results and Discussion

**Scheme 3:**
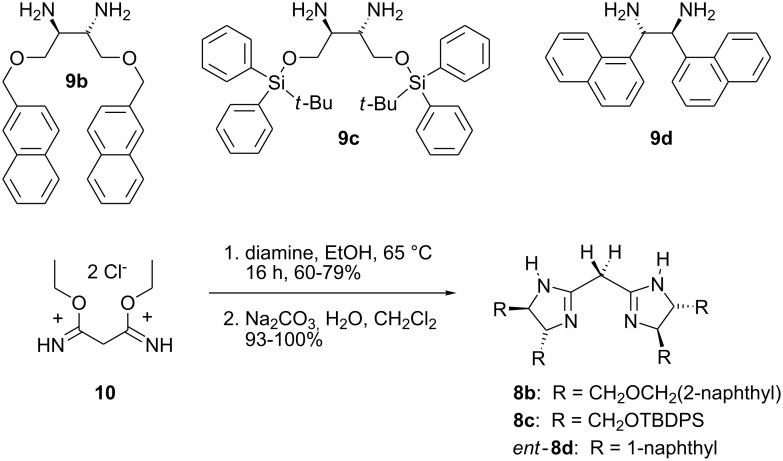
Synthesis of *C*_2_-symmetric bisamidines **8b**–**c** and *ent*-**8d**.

Analogous to the synthesis of compound **8a** [6], the other bisamidines were prepared as hydrochlorides in 60–79% yield from the corresponding chiral diamines **9** and bisimidate **10** in refluxing ethanol. Simple extraction in the presence of Na_2_CO_3_ afforded the neutral bases **8b**–**c** and *ent-***8d** in almost quantitative yield. The *S*,*S* configurated diamines **9b** and **9c** were prepared from L-(+)-tartaric acid (*R,R*) via the vicinal diazide using Saalfrank's procedure [9]. **9d** was purchased as the dihydrochloride salt and then deprotonated by aqueous sodium hydroxide. As an “artefact” of the sequence rule, the *S*,*S* configurated diamine **9d** leads to bisamidine *ent*-**8d** (Scheme 3).

The anthrones **1b** (R^1^: H; R^2^: Cl) and **1c** (R^1^: Cl; R^2^: H) resulted from regioselective reductions of 1,8-dichloroanthraquinone [10–11]. Aliphatic side chains of compounds **2** could be introduced by a Mitsunobu alkylation of maleimide [12]. Alternatively, substituted maleimides were prepared by reaction of maleic anhydride with the corresponding amines followed by ring closure [13–14].

**Figure 1:**
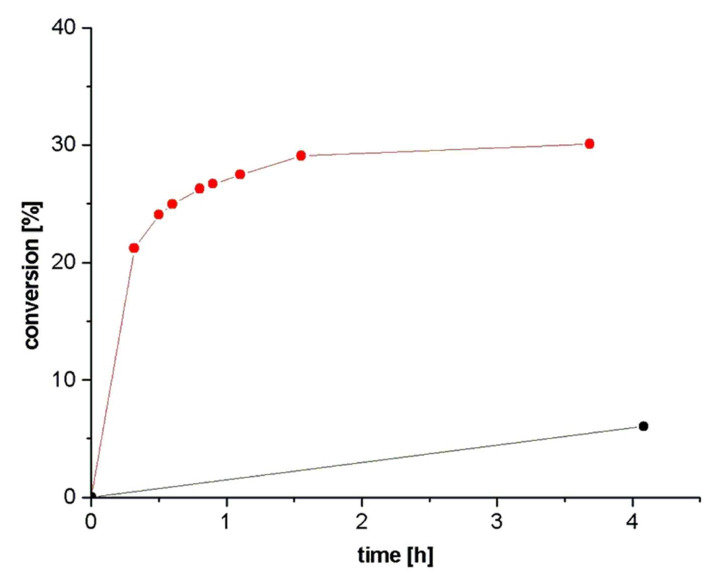
Kinetic measurements of **1a** with **2a** catalyzed by 5 mol% of **8a**·H^+^·TFPB^-^ (black line) and 1 mol% **8a** (free base; red line).

Cycloaddition kinetics of **1a** and **2a** was examined first by ^1^H NMR in CD_2_Cl_2_ at room temperature. In the absence of catalyst, no product could be observed after 4 days. 5 mol% of the bisamidinium salt **8a**·H^+^ with tetrakis(3,5-bis(trifluoromethyl)phenyl)borate (TFPB^-^) as weakly coordinating anion resulted in 7% yield of **3a** after 4 h. In contrast, only 1 mol% of the free Brønsted base **8a** led to a high rate increase in the first 30 min. After 90 min no further conversion was observed indicating product inhibition (Figure 1). Accordingly, the reaction runs best in the base-catalyzed mode. Compared to the bisoxazolines **7**, bisamidines **8** as stronger Brønsted bases induced much higher rates in all subsequent experiments.

In the next series of experiments, bisamidines **8a**–**c** and *ent*-**8d** were compared as catalysts of the cycloaddition forming **3a** from *N*-phenylmaleimide (**2a**) and anthrone (**1a**). Using 0.25 equiv of catalyst at room temperature, isolated yields between 71% and 86% were obtained after 30 min. The best enantioselectivity, albeit low, was induced by amidine **8c** (24% *ee*). As expected, in the presence of catalyst *ent*-**8d** product *ent*-**3a** was formed preferentially (Table 1).

**Table 1:** First evaluation step of chiral bisamidine catalysts.

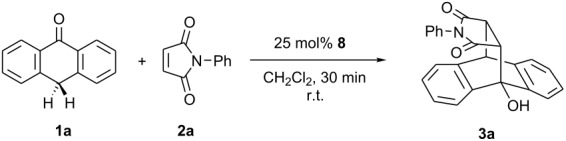			
entry^a^	catalyst	yield [%]^b^	*ee* [%]^c^

1	**8a**	86	11
2	**8b**	78	17
3	**8c**	85	24
4	*ent*-**8d**	71	−17^d^

^a^All reactions were carried out using 0.1 mmol maleimide **2a**, 1.1 equiv anthrone (**1a**) and 0.25 equiv of catalyst in 1 mL abs. dichloromethane at room temperature for 30 minutes. ^b^Isolated yield after column chromatography. ^c^The enantiomeric excess was determined by HPLC using a Chiralpak IA column. ^d^A negative *ee* stands for an excess of *ent*-**3a**.

In a solvent screening using 10 mol% of TBDPS-protected bisamidine **8c**, best results were obtained in dichloromethane (84% yield; 30% *ee*). Even higher yields were accessible in aromatic solvents, however, at the price of reduced stereoselectivity (Table 2).

**Table 2:** Influence of the solvent on the bisamidine catalyzed Diels-Alder reaction.

entry^a^	solvent	yield [%]^b^	*ee* [%]^c^

1	dichloromethane	84	30
2	chloroform	86	18
3	benzene	98	21
4	toluene	99	16
5	α,α,α-trifluorotoluene	99	13
6	dibutyl ether	89	11

^a^All reactions were carried out using 0.1 mmol maleimide **2a**, 1.1 equiv anthrone (**1a**) and 0.1 equiv of **8c** in 1 mL abs. solvent at room temperature for 60 minutes. ^b^Isolated yield after column chromatography. ^c^The enantiomeric excess was determined by HPLC using a Chiralpak IA column.

Lowering the reaction temperature from 23 to −20 °C (**8c**, dichloromethane) retarded the cycloaddition but did not change enantioselectivities. After extended reaction times, excellent yields were still observed. Up to 39% *ee* was finally obtained at −70 °C. However, such conditions resulted in lower yields, even with increased catalyst loads and further extended reaction times. Best results, 96% yield and 36% *ee* with only 10 mol% of catalyst, were found at −40 °C (Table 3).

**Table 3:** Influence of temperature on the Diels-Alder reaction.

entry^a^	reaction temperature [°C]	reaction time [h]	yield [%]^b^	*ee* [%]^c^

1	23	1	84	30
2	0	24	96	29
3	−20	24	98	31
4	−40	48	96	36
5	−70	96	71	39

^a^All reactions were carried out using 0.1 mmol maleimide **2a**, 1.1 equiv anthrone (**1a**) and 0.1 (entry 1–4) or 0.25 equiv (entry 5) of **8c** in 1 mL abs. dichloromethane. ^b^Isolated yield after column chromatography. ^c^Enantiomeric excess was determined by HPLC using Chiralpak IA column.

Having identified suitable experimental conditions, we explored the scope of the bisamidine-catalyzed Diels-Alder reaction. The results are summarized in Table 4. Both electron-donating and electron-withdrawing substituents were tolerated and furnished products in good to excellent yields and with moderate values of *ee*. A remarkable increase in enantioselectivity was observed using maleimide **2i**. The steric hindrance imposed by the large 2,6-diisopropylphenyl moiety of **2i** resulted in 76% *ee* at −70 °C but also lowered reaction rates.

**Table 4:** Scope of the Diels-Alder-reaction.

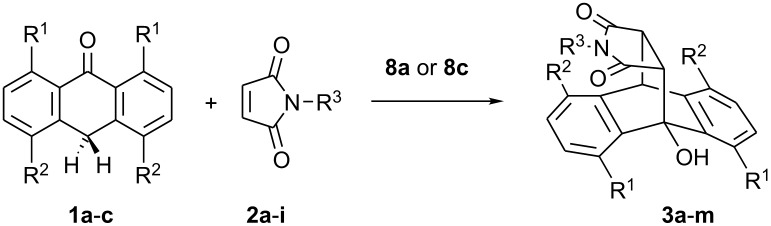						
entry^a^	**1** [R^1^, R^2^]	R^3^	condition^b^	**3**	yield [%]^c^	*ee* [%]^d^

1	**1a** [H, H,]	Ph (**2a**)	A	**3a**	96	36
2	**1b** [H, Cl]	**2a**	A	**3b**	95	41
3	**1a**	iPr (**2b**)	B	**3c**	74	26
4	**1a**	*t*-Bu (**2c**)	B	**3d**	45	30
5	**1a**	Cy (**2d**)	B	**3e**	83	42
6	**1c** [Cl, H]	**2d**	B	**3f**	90	19
7	**1a**	Bn (**2e**)	A	**3g**	95	20
8	**1a**	CHPh_2_ (**2f**)	A	**3h**	85	26
9	**1a**	4-Br-(C_6_H_4_)- (**2g**)	B	**3i**	70	13
10	**1a**	4-MeO-(C_6_H_4_)- (**2h**)	A	**3j**	82	32
11	**1a**	2,6-iPr_2_-(C_6_H_3_)- (**2i**)	B	**3k**	13	**76**
12	**1a**	**2i**	C	**3k**	65	51
13	**1c**	**2i**	C	**3l**	77	34
14	**1b**	**2i**	C	**3m**	76	54 (**96**)^e^

^a^All reactions were carried out using 0.1 mmol maleimide, 1.1 equiv anthrone in 1 mL abs. CH_2_Cl_2_. ^b^A = 10 mol% **8c**, −40 °C, 48 h; B = 25 mol% **8a**, −70 °C, 96 h; C = 25 mol% **8a**, r.t., 3 h. ^c^Isolated yield after column chromatography. ^d^The enantiomeric excess was determined by HPLC using a Chiralpak IA column. ^e^Recrystallized from 2-propanol/*n*-hexane.

**Figure 2:**
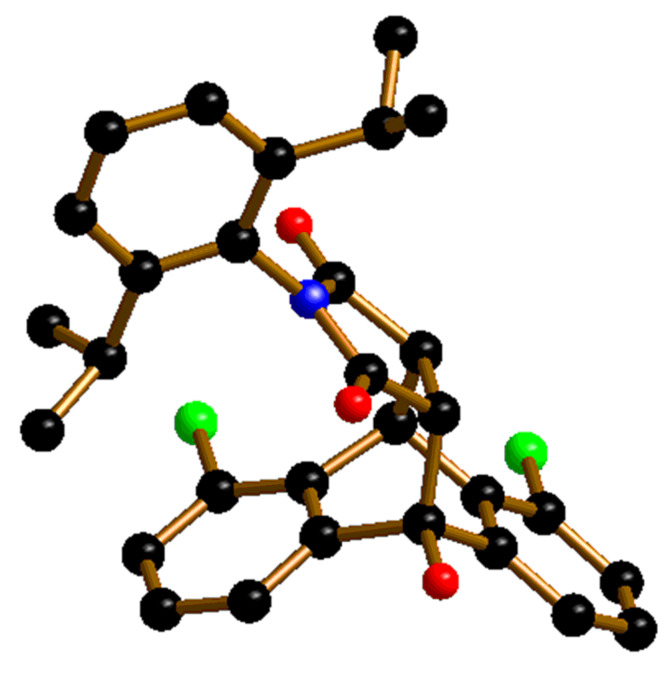
Molecular structure of **3m** (C: black; N: blue; O: red; Cl: green; hydrogen atoms are omitted for the sake of clarity).

Only 13% yield could be obtained under such conditions. Yields rose to 65% at room temperature (51% *ee*; entries 11 and 12). With other sterically hindered dienophiles such as *N*-*tert*-butylmaleimide (**2c**), the level of *ee* remained low (entry 4). The halogen-substituted anthrones **1b**–**c** did not react with **2i** at −70 °C. At room temperature, however, **1b** and **2i** were efficiently transformed into **3m** by catalyst **8a** with 76% yield and 54% *ee*. A single recrystallisation step afforded an almost enantiopure product (96% *ee*). The *R*,*R* configuration of compound **3m** was determined by anomalous X-ray diffraction using a single crystal of **3m** with 96% *ee* (Figure 2).

**Scheme 4:**
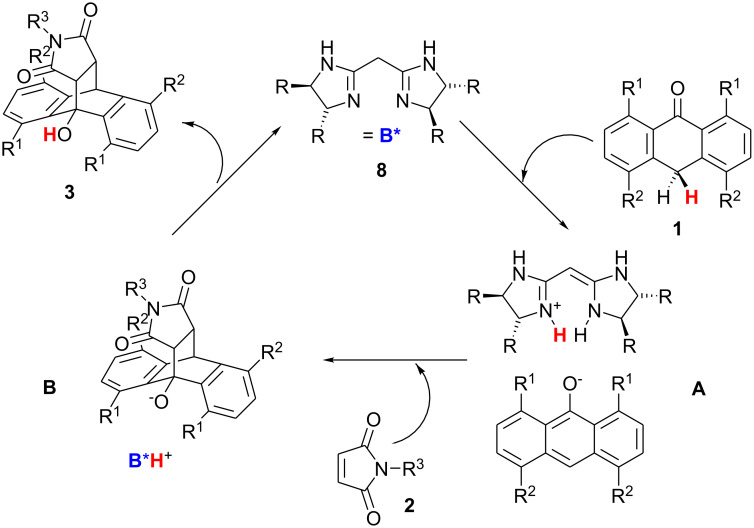
Proposed mechanism of the Diels-Alder reaction.

A mechanistic rationalisation is proposed in Scheme 4. The catalyst deprotonates the anthrone in the initial step. This assumption is supported by the p*K*_a_ values of compounds **2a** (10, [7–8]) and **8**·H^+^ (~11, [6]). Furthermore, the appearance of the yellow color of enolates (**1**·H^+^) shows significant proton transfer when bisamidine **8a** is added to anthrones **1a**, **1b**, or **1c**. A chiral contact ion pair **A** is formed and controls the stereochemical course of the Diels-Alder reaction with maleimides. In the last step, the catalyst-product-complex **B** dissociates and regenerates the unprotonated bisamidine.

## Conclusion

*C*_2_-symmetric bisamidines were shown to be potent chiral Brønsted base catalysts for the Diels-Alder reaction of *N*-substituted maleimides and anthrones. Compared to bisoxazolines **7**, much shorter reaction times under comparable conditions were sufficient with the more basic bisamidine catalysts **8** (~50-fold [5]). The higher intrinsic reactivity of the bisamidines allowed to run the reactions at lower temperatures. In both groups of catalysts, the phenyl substituted species induced the lowest enantioselectivities. Bisamidine **8a** performed better than the corresponding bisoxazoline. Increasing the size of substituents in catalysts **8b**–**d** also improved stereoselectivities, but not to high levels. This may be due to the flexible nature of the substituents present in bisamidines **8b** and **8c**. It is instructive, therefore, to compare with the bisoxazolines **7**. By far the best enantioselectivities were observed in this series with the *t*-Bu derivative (47% *ee* versus 3% for the phenyl analogue in the reaction of **1a** and **2a**). Keeping in mind that even the less selective bisamidine **8a** could induce up to 76% *ee* in favorable cases, replacing the phenyl moieties of **8a** by *t*-Bu is an attractive option for future studies on bisamidine-mediated organocatalytic transformations.

## Supporting Information

File 1 Supporting information features characterisation data and copies of ^1^H- and ^13^C-NMR spectra of anthrones **1**, maleimides **2**, Diels-Alder adducts **3**, bisamidine hydrochlorides **8b**–**d**·H^+^·Cl^-^, neutral bisamidines **8b**–**d** and diamines **9b**–**c**, plus copies of chromatograms obtained with chiral columns.

File 2 X-Ray data of compound **3k**

File 3 X-Ray data of compound **3m**
