# Supplementary material for: C2-symmetric bisamidines: Chiral Brønsted bases catalysing the Diels-Alder reaction of anthrones
Source: Beilstein J Org Chem. 2008 Aug 7;4:28. doi: 10.3762/bjoc.4.28 (PMC2533436; doi:10.3762/bjoc.4.28)
Supplement: File 1 — Supporting information features characterisation data and copies of 1H- and 13C-NMR spectra of anthrones 1, maleimides 2, Diels-Alder adducts 3, bisamidine hydrochlorides 8b–d·H+·Cl-, neutral bisamidines 8b–d and diamines 9b–c, plus copies of chromatograms obtained with chiral columns. [file Beilstein_J_Org_Chem-04-28-s001.doc]

**Supporting Information**

# *C*_2_-symmetric bisamidines: Chiral Brønsted bases catalysing the Diels-Alder reaction of anthrones

Deniz Akalay, Gerd Dürner, Jan W. Bats and Michael W. Göbel*

Address: Johann Wolfgang Goethe University Frankfurt, Institute of Organic Chemistry and Chemical Biology, Max-von-Laue-Str. 7, D-60438 Frankfurt am Main, Germany.

Email: Michael W. Göbel* - M.Goebel@chemie.uni-frankfurt.de

* Corresponding author

Table of Contents:

S2 Experimental Section

S2 General Methods

S2 Chiral Diamines **9**

S3 *C*_2_-symmetric bisamidines **8**

S6 Anthrone derivatives **1**

S6 *N*-substituted maleimides **2**

S8 General procedure for Diels-Alder reaction of **1** with **2**.

S8 Diels-Alder adducts **3**

S15 General procedure for kinetics of reaction **1a** with **2a**.

S16 Results for optimal reaction conditions

S17 Spectroscopic Data of Compounds

S44 Copies of chromatograms on chiral column

S59 X-Ray Data of **3k** and **3m**

S62 References

**Experimental section**

**General Methods** All NMR spectra were recorded on a 250 MHz, 300 MHz or 400 MHz spectrometer. ^1^H chemical shifts (δ) are quoted in parts per million downfield to tetramethylsilane, relative to CDCl_3_ (7.26 ppm) or DMSO (2.50 ppm) against an internal deuterium lock; multiplicities are indicated as s for singlet, d-doublet, dd-double doublet, t-triplet, sp-septet, m-multiplet, br s-broad singlet; *J* in Hz; ^13^C chemical shifts (δ) are reported with complete proton decoupling and are quoted in parts per million downfield to tetramethylsilane, relative to CDCl_3_ (t, 77.0 ppm) or DMSO (septet, 39.43 ppm) against an internal deuterium lock. FT-IR spectra were recorded as liquid films on NaCl plates or as KBr pellets (KBr); peaks are reported in cm^-1^, intensities are classified as strong (s), medium (m) and weak (w). Melting points are uncorrected. Analytical thin layer chromatography (TLC) with F_254_ indicator were used with visualization by UV light (254 nm).

Anhydrous dichloromethane and ethanol were stored over molecular sieves, anthrone (**1a**), maleimides **2a**, **2d**, **2e**, and chiral diamine **9d***2HCl were purchased and used without further purification. CDCl_3_ was stored over molecular sieves and NaHCO_3_.

**(2*S*,3*S*)-1,4-Bis(2-naphthylmethoxy)-2,3-diaminobutane (9b)**. The synthesis of **9b** was carried out as in the reported procedure (32% over 9 steps) [1]. Colorless crystals; m.p. 110-111 °C (Lit. 100-102 °C) [1]; ^1^H NMR (250 MHz, CDCl_3_) δ 1.42 (br s, 4 H), 3.02-3.09 (m, 2 H), 3.46 (dd, *J*_1_ = 6.5 Hz, *J*_2_ = 9.3 Hz, 2 H), 3.55 (dd, *J*_1_ = 4.4 Hz, *J*_2_ = 9.1 Hz, 2 H), 4.66 (s, 4 H), 7.42-7.51 (m, 6 H), 7.75 (d, *J* = 0.5 Hz, 2H), 7.79-7.85 (m, 6 H); ^13^C NMR (63 MHz, CDCl_3_) δ 52.70, 73.39, 73.62, 125.68, 125.87, 126.11, 126.45, 127.67, 127.83, 128.20, 132.96, 133.21, 135.65; IR (KBr) 3367 (w), 3292 (w), 3049 (w), 2899 (m), 2855 (m), 1600 (w), 1506 (w), 1477 (w), 1439 (w), 1407 (w), 1347 (m), 1274 (w), 1246 (w), 1176 (w), 1119 (s), 1062 (w), 1042 (w), 1008 (w), 998 (w), 950 (w), 926 (w), 902 (m), 890 (w), 864 (s), 820 (s), 752 (s), 731 (w) cm^-1^; optical rotation: [α]^D^_24_ = +12.7 (*c* = 1.2 CHCl_3_, Lit. [α]^D^_24_ = -10.7* (*c* = 2.5 CHCl_3_) [1]); Anal. Calcd for C_26_H_28_N_2_O_2_: C, 77.97; H, 7.05; N, 6.99; Found: C, 77.95; H, 7.10; N, 6.82. *Note: The direction of the optical rotation deviates from reference [1]. However, the optical rotations of all intermediates leading from l-(+)-tartaric acid (*R,R*) to diamine **9b** agree well with data given in [1].

**(2*S*,3*S*)-1,4-Bis(*tert*-butyldiphenylsilyloxy)-2,3-diaminobutane (9c)**. The synthesis of **9c** was carried out as in the reported procedure (29% over 9 steps) [1]. Colorless oil; ^1^H NMR (250 MHz, CDCl_3_) δ 1.05 (s, 18 H), 1.66 (br s, 4 H), 2.89-2.96 (m, 2 H), 3.55 (dd, *J*_1_ = 6.0 Hz, *J*_2_ = 10.0 Hz, 2 H), 3.63 (dd, *J*_1_ = 6.0 Hz, *J*_2_ = 10.0 Hz, 2 H), 7.31-7.45 (m, 12 H), 7.60-7.65 (m, 8 H); ^13^C NMR (63 MHz, CDCl_3_) δ 19.25, 26.90, 53.81, 66.72, 127.71, 129.71, 133.39, 133.41, 135.54; IR (Film) 3384 (w), 3071 (m), 3049 (m), 2958 (s), 2950 (s), 2891 (m), 2857 (s), 1589 (w), 1472 (m), 1428 (s), 1390(w), 1361 (w), 1261 (w), 1187 (w), 1112 (s), 1007 (w), 938 (w), 824 (m), 740 (m), 701 (s) cm^-1^; optical rotation: [α]^D^_20_ = +0.8 (*c* = 1.0 MeOH); ESI^+^ Mass Spectra m/z Calcd for C_36_H_49_N_2_O_2_Si_2_^+^: 597.3 (M+H^+^). Found: 597.5.

**General Procedure for the synthesis of *C*_2_-symmetric bisamidines 8 as hydrochloride**

After addition of 0.55 equiv malonodiimic acid diethyl ester dihydrochloride **10** [2] to a solution of diamine **9** in abs. ethanol (~ 0.1 M) the reaction mixture was stirred at 65 °C for 16 h. The crude reaction mixture was concentrated in vacuo, adsorbed on silica gel and purified by column chromatography (ethyl acetate/methanol eluent), which afforded bisamidine **8** as the hydrochloride salt.

The synthesis of **8a***HCl was carried out as in the reported procedure [2].

**8b***HCl Yield 79%; yellowish foam; m.p. 63-66 °C; ^1^H NMR (250 MHz, DMSO) δ 3.51 (br s, 8 H), 3.88 (br s, 4 H), 4.10 (s, 1 H), 4.69 (br s, 8 H), 7.43-7.52 (m, 12 H), 7.80-8.06 (m, 20 H); IR (KBr) 3171 (m), 3050 (m), 2857 (m), 1589 (s), 1508 (m), 1368 (w), 1340 (w), 1271 (w), 1212 (w), 1171 (w), 1124 (m), 1102 (m), 952 (w), 894 (w), 856 (w), 816 (m), 751 (m) cm^-1^; optical rotation: [α]^D^_20_ = -15.9 (*c* = 1.0 MeOH); Anal. Calcd for C_55_H_53_ClN_4_O_4_: C, 75.97; H, 6.14; N, 6.44; Found: C, 75.77; H, 5.93; N, 6.66.

**8c***HCl Yield 60%; yellowish foam; m.p. 68-71 °C; ^1^H NMR (250 MHz, DMSO) δ 0.95 (s, 36 H), 3.64 (br s, 8 H), 3.94 (br s, 4 H), 4.15 (s, 1 H), 7.35-7.48 (m, 24 H), 7.59-7.62 (m, 16 H), 7.83 (br s, 4 H); IR (KBr) 3178 (w), 3049 (w), 2929 (m), 2855 (m), 1588 (s), 1472 (w), 1427 (m), 1389 (w), 1362 (w), 1113 (s), 1007 (w), 937 (w), 883 (w), 823 (w), 739 (w), 701 (s) cm^-1^; optical rotation: [α]^D^_20_ = +22.3 (*c* = 1.2 MeOH); ESI Mass Spectra m/z Calcd for C_75_H_93_N_4_O_4_Si_4_^+^: 1225.6 (M-Cl^-^). Found: 1226.0.

*ent*-**8d***HCl Yield 75%; yellowish foam; m.p. 248-250 °C (decomp.); ^1^H NMR (400 MHz, DMSO) δ 4.73 (s, 1 H), 5.80 (s, 4 H), 7.23 (t, J = 7.6 Hz, 4 H), 7.46 (t, J = 7.6 Hz, 4 H) 7.53 (d, J = 8.4 Hz, 4 H), 7.66 (t, J = 7.6 Hz, 4 H), 7.81 (t, J = 6.0 Hz, 4 H), 7.99 (t, J = 7.6 Hz, 8 H), 8.70 (br s, 4 H); IR (KBr) 3404 (w), 3133 (w), 3047 (m), 1584 (s), 1511 (m), 1490 (w), 1396 (w), 1383 (w), 1362 (w), 1320 (w), 1306 (w), 1260 (w), 1212 (w), 1166 (w), 1102 (m), 800 (m), 798 (m), 775 (s), 735 (w) cm^-1^; optical rotation: [α]^D^_20_ = +227.2 (*c* = 0.5 MeOH); Anal. Calcd for C_47_H_37_ClN_4_: C, 81.43; H, 5.38; N, 8.08; Found: C, 81.22; H, 5.52; N, 8.04.

**General Procedure for the deprotonation of 8*HCl**

A two-phase mixture of **8***HCl in CH_2_Cl_2_ (~ 0.05 M) and saturated Na_2_CO_3_ aqueous solution (1:1) was stirred for 30 minutes. The separated aqueous phase was extracted with dichloromethane. The combined organic phase was dried over MgSO_4_ and concentrated in vacuo to give neutral bisamidine **8**.

The synthesis of **8a** was carried out as in the reported procedure [2].

**8b** Yield 93%; yellowish foam; m.p. 127-128 °C; ^1^H NMR (250 MHz, CDCl_3_) δ 1.68 (br s, 2 H), 3.24 (s, 2 H), 3.45 (br s, 8 H), 3.82 (br s, 4 H), 4.60 (br s, 4 H), 7.36-7.48 (m, 12 H), 7.68-7.82 (m, 16 H); IR (KBr) 3050 (w), 2919 (s), 2850 (s), 1579 (s), 1560 (m), 1508 (w), 1450 (m), 1124 (s), 1095 (s), 1018 (s), 854 (w), 817 (w), 749 (w) cm^-1^; optical rotation: [α]^D^_20_ = +93.2 (*c* = 1.0 MeOH); Anal. Calcd for C_55_H_52_N_4_O_4_: C, 79.30; H, 6.29; N, 6.73; Found: C, 79.24; H, 6.34; N, 6.59.

**8c** Yield 95%; yellowish foam; m.p. 48-51 °C; ^1^H NMR (250 MHz, CDCl_3_) δ 0.44 (br s, 2 H), 0.99 (s, 36 H), 3.11 (s, 2 H), 3.59-3.94 (m, 12 H), 7.27-7.41 (m, 24 H), 7.60-7.65 (m, 16 H); IR (KBr) 3070 (w), 3048 (w), 2929 (m), 2856 (m), 1618 (w), 1472 (w), 1427 (m), 1389 (w), 1362 (w), 1113 (s), 998 (w), 823 (w), 739 (w), 700(s) cm^-1^; optical rotation: [α]^D^_20_ = +50.0 (*c* = 1.0 MeOH); Anal. Calcd for C_75_H_92_N_4_O_4_Si_4_: C, 73.48; H, 7.56; N, 4.57; Found: C, 73.22; H, 7.65; N, 4.41.

*ent*-**8d** Yield 100%; yellowish foam; m.p. 138-140 °C; ^1^H NMR (250 MHz, CDCl_3_) δ 3.85 (br s, 2 H), 4.44 (br s, 2 H), 5.65 (s, 4 H), 7.05-7.10 (m, 4 H), 7.32-7.47 (m, 12 H), 7.60-7.63 (m, 4 H), 7.75-7.82 (m, 12 H); IR (KBr) 3396 (w), 3046 (m), 2930 (w), 1617 (s), 1596 (s), 1510 (m), 1458 (m), 1396 (m), 1383 (w), 1356 (w), 1312 (w), 1257 (w), 1216 (w), 1165 (w), 1082 (w), 1002 (w), 858 (w) cm^-1^; optical rotation: [α]^D^_20_ = +134.0 (*c* = 0.25 MeOH); ESI Mass Spectra m/z Calcd for C_47_H_36_N_4_^+^: 657.3 (M+H^+^). Found: 657.4. Calcd for ([M+2H^+^]/2): 329.2, Found: 329.3. Calcd for ([M+2H^+^-CH_2_]/2): 322.1, Found: 323.0.

The synthesis of **1b** and **1c** was carried out as in the reported procedure [3,4].

**4,5-dichloro-10*H*-anthracen-9-one (1b)**. Yield: 53%; yellow crystals; m.p. 197-198 °C (acetone, Lit. 194 °C) [3]; ^1^H NMR (250 MHz, CDCl_3_) δ 4.26 (s, 2 H), 7.47 (t, *J* = 7.9Hz, 2 H), 7.71 (dd, *J*_1_ = 1.4 Hz, *J*_2_ = 7.9Hz, 2 H), 8.30 (dd, *J*_1_ = 1.4 Hz, *J*_2_ = 7.9Hz, 2 H); ^13^C NMR (63 MHz, CDCl_3_) δ 29.45, 126.36, 128.07, 132.83, 133.70, 134.16, 137.30, 182.69; IR (KBr) 3081 (w), 1659 (s), 1591 (s), 1560 (w), 1458 (w), 1442 (w), 1391 (m), 1339 (w), 1312 (s), 1284 (w), 1168 (w), 1135 (s), 1071 (w), 961 (w), 915 (w), 871(m), 820 (m), 786 (w), 743 (s) cm^-1^; Calcd for C_14_H_8_Cl_2_O: C, 63.91; H, 3.06; Found: C, 63.65; H, 3.11.

**1,8-dichloro-10*H*-anthracen-9-one (1c)**. Yield 20%; yellow crystals; m.p. 166-168 °C (dichloromethane/n-hexane, Lit. 167-168 °C) [4]; ^1^H NMR (250 MHz, CDCl_3_) δ 4.20 (s, 2 H), 7.29-7.46 (m, 6 H); ^13^C NMR (63 MHz, CDCl_3_) δ 33.86, 126.25, 130.23, 131.78, 132.01, 133.51, 140.80, 183.45; IR (KBr) 3069 (w), 2861 (w), 1676 (s), 1592 (s), 1560 (w), 1508 (w), 1454 (w), 1437 (m), 1406 (w), 1334 (w), 1296 (w), 1255 (m), 1190 (w), 1162 (w), 1140 (w), 944 (w), 916 (w), 882 (w), 769 (s), 753 (s), 692 (w), 681 (m) cm^-1^; Anal. Calcd for C_14_H_8_Cl_2_O: C, 63.91; H, 3.06; Found: C, 64.00; H, 3.08.

***N*-substituted maleimides 2**. The synthesis of **2** was carried out as in the reported procedure [5-7].

**2b** Yield 67%; colorless oil; ^1^H NMR (250 MHz, CDCl_3_) δ 1.38 (d, *J* = 6.8 Hz, 6 H), 4.32 (sp, *J* = 6.9 Hz, 1H), 6.61 (s, 2 H); ^13^C NMR (63 MHz, CDCl_3_) δ 20.11, 42.96, 133.93, 170.80; IR (film) 3456 (m), 3102 (m), 2978 (m), 2940 (m), 2882 (m), 1765 (m), 1702 (s), 1596 (w), 1459 (m), 1407 (s), 1385 (s), 1367 (s), 1294 (w), 1207 (m), 1182 (w), 1117 (m), 1021 (m), 988 (m), 879 (w), 829 (s) cm^-1^. Mass spectra m/z calcd for C_7_H_10_NO_2_^+^: 140.1 (M+H^+^). Found: 140.1, m/z calcd for C_7_H_9_NO_2_^+^: 139.1 (M^+^). Found: 139.1, m/z calcd for C_6_H_6_NO_2_^+^: 124.0 (M^+^-CH_3_). Found: 124.1, m/z calcd for C_4_H_4_NO_2_^+^: 98.0 (Maleimide+H^+^). Found: 98.0, m/z calcd for C_4_H_2_NO_2_^+^: 96.0 (M^+^-*^i^*Pr). Found: 96.1.

**2c** Yield 14%; colorless oil; ^1^H NMR (250 MHz, CDCl_3_) δ 1.58 (s, 9 H), 6.51 (s, 2 H); ^13^C NMR (63 MHz, CDCl_3_) δ 28.87, 57.37, 133.89, 172.15; IR (film) 3454 (w), 3104 (w), 2977 (m), 1707 (s), 1603 (w), 1459 (m), 1390 (w), 1351 (s), 1260 (w), 1211 (m), 1140 (s), 1079 (w), 1003 (w), 984 (m), 829 (m), 692 (s) cm^-1^; Anal. Calcd for C_8_H_11_NO_2_: C, 62.73; H, 7.24; N, 9.14; Found: C, 62.53; H, 7.42; N, 9.09.

**2f** Yield 28%; colorless crystals; m.p. 144-146 °C (MeOH, Lit. 139-141 °C) [8]; ^1^H NMR (250 MHz, CDCl_3_) δ 6.51 (s, 1 H), 6.71 (s, 2 H), 7.27-7.38 (m, 10 H); ^13^C NMR (63 MHz, CDCl_3_) δ 57.61, 127.74, 128.40, 128.52, 134.16, 138.05, 170.30; IR (KBr) 3096 (m), 3026 (m), 1698 (s), 1598 (w), 1491 (m), 1448 (w), 1399 (s), 1362 (s), 1314 (w), 1260(w), 1203 (w), 1150 (s), 1112 (w), 1082 (w), 1026 (m), 918 (w), 868 (m), 832 (s), 796 (m), 752 (m), 731 (m), 699 (s) cm^-1^; Anal. Calcd for C_17_H_13_NO_2_: C, 77.55; H, 4.98; N, 5.32; Found: C, 77.43; H, 5.14; N, 5.21.

**2g** Yield 55%; yellow crystals; m.p. 122-124 °C (2-propanol, Lit. 118-120 °C) [9]; ^1^H NMR (250 MHz, CDCl_3_) δ 6.86 (s, 2 H), 7.23-7.29 (m, 2 H), 7.56-7.62 (m, 2H); ^13^C NMR (63 MHz, CDCl_3_) δ 121.57, 127.33, 130.26, 132.27, 134.28, 169.03; IR (KBr) 1708 (s), 1560 (w), 1490 (s), 1389 (m), 1212 (w), 1153 (m), 1065 (w), 1013 (w), 950 (w), 827 (m), 733 (w), 712 (m), 687 (m) cm^-1^; Anal. Calcd for C_10_H_6_BrNO_2_: C, 47.65; H, 2.40; N, 5.56; Found: C, 47.74; H, 2.47; N, 5.48.

**2h** Yield 35%; yellow crystals; m.p. 146-148 °C (EtOH, Lit. 148 °C) [10]; ^1^H NMR (250 MHz, CDCl_3_) δ 3.83 (s, 3 H), 6.83 (s, 2 H), 6.95-7.01 (m, 2 H), 7.20-7.26 (m, 2 H); ^13^C NMR (63 MHz, CDCl_3_) δ 55.47, 114.46, 123.73, 127.55, 134.10, 159.14, 169.78; IR (KBr) 3109 (w), 2966 (w), 2836 (w), 1707 (s), 1607 (w), 1587 (w), 1510 (s), 1450 (w), 1441 (w), 1414 (m), 1400 (m), 1304 (m), 1249 (s), 1180 (w), 1166 (m), 1159 (m), 1106 (w), 1055 (w), 1029 (m), 962 (w), 955 (w), 940 (w), 838 (m), 828 (m), 800 (w), 722 (w), 687 (w) cm^-1^; Anal. Calcd for C_11_H_9_NO_3_: C, 65.02; H, 4.46; N, 6.89; Found: C, 64.90; H, 4.55; N, 6.78.

**2i** Yield 53%; yellow crystals; m.p. 114-116 °C (n-hexane, Lit. 111-114 °C) [7]; ^1^H NMR (250 MHz, CDCl_3_) δ 1.16 (d, *J* = 7.0 Hz, 12 H), 2.62 (sp, *J* = 6.9 Hz, 2 H), 6.88 (s, 2 H), 7.24-7.27 (m, 2 H), 7.40-7.46 (m, 1 H); ^13^C NMR (63 MHz, CDCl_3_) δ 23.91, 29.29, 123.97, 126.17, 130.21, 134.26, 147.46, 170.50; IR (KBr) 3468 (w), 3091 (w), 2967 (m), 2930 (w), 2872 (w), 1710 (s), 1459 (m), 1391 (m), 1375 (m), 1250 (w), 1157 (w), 1157 (m), 1056 (w), 1033 (w), 950 (w), 937 (w), 834 (m), 802 (m), 755 (w), 690 (m) cm^-1^; Anal. Calcd for C_16_H_19_NO_2_: C, 74.68; H, 7.44; N, 5.44; Found: C, 74.54; H, 7.24; N, 5.26.

**General procedure for the Diels-Alder reaction of anthrone derivatives 1 with *N*-substituted maleimides 2**. 0.1 mmol of maleimide **2**, 1.1 equiv of corresponding anthrone **1** and catalyst **8** were cooled to the indicated temperature. After addition of temperated CH_2_Cl_2_ (abs., 1 mL) the solution was stirred as described in table 4. The crude reaction mixture was purified by column chromatography (ethyl acetate/n-hexane eluent) to afford **3** as colorless crystalline solid. The racemic compound was prepared with triethylamine (10 μl) instead of bisamidine. After 30 minutes the crude product was also purified by flash column chromatography.

**3a** Yield 96%; m.p. 199-201 °C (Lit. 208-209°C) [11]; ^1^H NMR (250 MHz, CDCl_3_) δ 3.28 (d, *J* = 8.5 Hz, 1 H), 3.49 (dd, *J*_1_ = 3.6 Hz, *J*_2_ = 8.6 Hz, 1 H), 4.54 (s, 1 H), 4.84 (d, *J* = 3.5 Hz, 1 H), 6.45-6.52 (m, 2 H), 7.20-7.36 (m, 8 H), 7.40-7.43 (m, 1 H), 7.55-7.58 (m, 1 H), 7.72-7.75 (m, 1 H); ^13^C NMR (63 MHz, CDCl_3_) δ 44.79, 47.66, 50.78, 77.30, 120.88, 121.09, 123.76, 124.69, 126.26, 126.84, 126.92, 127.24, 127.35, 128.97, 129.11, 130.86, 136.62, 138.83, 140.92, 142.26, 175.59, 177.11; IR (KBr) 3416 (m), 3070 (w), 2961 (w), 1773 (w), 1699 (s), 1596 (w), 1494 (m), 1458 (m), 1381 (s), 1296 (w), 1266 (w), 1245 (w), 1175 (s), 1136 (w), 1073 (w), 1056 (w), 1028 (w), 990 (w), 962 (w), 946 (w), 922 (w), 846 (w), 786 (w), 770 (s), 756 (m), 724 (w), 690 (w) cm^-1^; Anal. Calcd for C_24_H_17_NO_3_: C, 78.46; H, 4.66; N, 3.81; Found: C, 78.40; H, 4.73; N, 3.61. HPLC: CHIRALPAK IA, 250 x 4.6 mm (l x i.d.), flow-rate 0.7 mL/min, UV-254 nm, n-hexane/isopropyl alcohol 10 + 3 + 20% CH_2_Cl_2_, t_major_: 12.1 min, t_minor_: 10.6 min, 36% *ee*.

**3b** Yield 95%; m.p. 234-236 °C; ^1^H NMR (250 MHz, CDCl_3_) δ 3.21 (d, *J* = 8.8 Hz, 1 H), 3.46 (dd, *J*_1_ = 3.8 Hz, *J*_2_ = 8.8 Hz, 1 H), 4.59 (s, 1 H), 5.88 (d, *J* = 3.8 Hz, 1 H), 6.50-6.54 (m, 2 H), 7.16-7.30 (m, 7 H), 7.40-7.43 (m, 1 H), 7.56-7.60 (m, 1 H); ^13^C NMR (63 MHz, CDCl_3_) δ 37.64, 46.16, 49.88, 77.36, 119.59, 119.98, 126.10, 127.60, 128.07, 128.16, 128.34, 129.08, 129.22, 130.12, 130.71, 131.02, 133.94, 135.51, 143.12, 144.48, 174.24, 176.69; IR (KBr) 3502 (m), 3070 (w), 2938 (w), 1776 (w), 1702 (s), 1597 (w), 1578 (w), 1499 (m), 1455 (s), 1425 (w), 1388 (s), 1278 (w), 1236 (m), 1188 (s), 1173 (s), 1112 (w), 1058 (w), 1004 (w), 958 (w), 932 (w), 893 (w), 874 (w), 829 (w), 781 (m), 771 (m), 749 (m) cm^-1^; Anal. Calcd for C_24_H_15_Cl_2_NO_3_: C, 66.07; H, 3.47; N, 3.21; Found: C, 65.93; H, 3.69; N, 3.03. HPLC: CHIRALPAK IA, 250 x 4.6 mm (l x i.d.), flow-rate 0.7 mL/min, UV-254 nm, n-hexane/isopropyl alcohol 10 + 3 + 20% CH_2_Cl_2_, t_major_: 14.6 min, t_minor_: 10.8 min, 41% *ee*.

**3c** Yield 74%; m.p. 183-185 °C; ^1^H NMR (250 MHz, CDCl_3_) δ 0.83 (d, *J* = 7.0 Hz, 3 H), 0.87 (d, *J* = 7.0 Hz, 3 H), 3.01 (d, *J* = 8.5 Hz, 1 H), 3.23 (dd, *J*_1_ = 3.6 Hz, *J*_2_ = 8.6 Hz, 1 H), 3.90 (sp, *J* = 7.0 Hz, 1 H), 4.58 (s, 1 H), 4.71 (d, *J* = 3.5 Hz, 1 H), 7.11-7.29 (m, 5 H), 7.35-7.38 (m, 1 H), 7.48-7.51 (m, 1 H), 7.66-7.70 (m, 1 H); ^13^C NMR (63 MHz, CDCl_3_) δ 18.35, 18.48, 43.63, 44.64, 47.01, 50.00, 77.18, 120.72, 121.05, 123.66, 124.59, 126.67, 126.77, 126.97, 127.08, 136.65, 139.08, 140.99, 142.57, 176.48, 178.16; IR (KBr) 3406 (w), 3044 (w), 2971 (w), 1765 (w), 1690 (s), 1459 (m), 1400 (w), 1364 (m), 1296 (w), 1262 (w), 1230 (m), 1205 (m), 1168 (w), 1144 (w), 1123 (m), 1001 (w), 922 (w), 875 (w), 768 (m), 751 (m), 716 (w) cm^-1^; Anal. Calcd for C_21_H_19_NO_3_: C, 75.66; H, 5.74; N, 4.20; Found: C, 75.66; H, 5.81; N, 4.08. HPLC: CHIRALPAK IA, 250 x 4.6 mm (l x i.d.), flow-rate 0.7 mL/min, UV-254 nm, n-hexane/isopropyl alcohol 10 + 3 + 20% CH_2_Cl_2_, t_major_: 6.9 min, t_minor_: 7.8 min, 26% *ee*.

**3d** Yield 45%; m.p. 217-219 °C; ^1^H NMR (250 MHz, CDCl_3_) δ 1.13 (s, 9 H), 2.93 (d, *J* = 9.0 Hz, 1 H), 3.14 (dd, *J*_1_ = 3.5 Hz, *J*_2_ = 9.0 Hz, 1 H), 4.68 (d, *J* = 3.8 Hz, 1 H), 4.71 (s, 1 H), 7.13-7.29 (m, 5 H), 7.34-7.37 (m, 1 H), 7.49-7.53 (m, 1 H), 7.65-7.69 (m, 1 H); ^13^C NMR (63 MHz, CDCl_3_) δ 27.67, 44.83, 46.92, 49.83, 68.63, 77.23, 120.71, 121.14, 123.59, 124.60, 126.59, 126.69, 126.87, 126.97, 136.77, 139.15, 141.17, 142.64, 177.65, 179.46; IR (KBr) 3490 (w), 2976 (w), 1763 (w), 1686 (s), 1560 (w), 1508 (w), 1458 (m), 1347 (m), 1260 (w), 1156 (m), 1008 (w), 880 (w), 769 (m), 753 (m) cm^-1^; Anal. Calcd for C_22_H_21_NO_3_: C, 76.06; H, 6.09; N, 4.03; Found: C, 75.84; H, 6.23; N, 3.84. HPLC: CHIRALPAK IA, 250 x 4.6 mm (l x i.d.), flow-rate 0.7 mL/min, UV-254 nm, n-hexane/isopropyl alcohol 10 + 3 + 20% CH_2_Cl_2_, t_major_: 6.6 min, t_minor_: 7.2 min, 30% *ee*.

**3e** Yield 83%; m.p. 226-229 °C; ^1^H NMR (250 MHz, CDCl_3_) δ 0.75-1.73 (m, 10 H), 3.01 (d, *J* = 8.5 Hz, 1 H), 3.23 (dd, *J*_1_ = 3.6 Hz, *J*_2_ = 8.6 Hz, 1 H), 3.43-3.59 (m, 1 H), 4.59 (s, 1 H), 4.71 (d, *J* = 3.5 Hz, 1 H), 7.11-7.29 (m, 5 H), 7.34-7.38 (m, 1 H), 7.47-7.50 (m, 1 H), 7.66-7.69 (m, 1 H); ^13^C NMR (63 MHz, CDCl_3_) δ 24.84, 25.56, 27.84, 27.96, 44.67, 46.98, 49.97, 51.48, 77.18, 120.70, 121.03, 123.65, 124.57, 126.66, 126.76, 126.96, 127.07, 136.62, 139.09, 140.96, 142.58, 176.58, 178.27; IR (KBr) 3422 (m), 3074 (w), 2930 (w), 2858 (w), 1764 (w), 1685 (s), 1458 (m), 1396 (w), 1376 (m), 1346 (m), 1296 (w), 1264 (w), 1242 (w), 1200 (m), 1183 (m), 1147 (w), 1071 (w), 1031 (w), 994 (w), 923 (w), 896 (w), 876 (w), 770 (m), 734 (w) cm^-1^; Anal. Calcd for C_24_H_23_NO_3_: C, 77.19; H, 6.21; N, 3.75; Found: C, 77.16; H, 6.30; N, 3.54. HPLC: CHIRALPAK IA, 250 x 4.6 mm (l x i.d.), flow-rate 0.7 mL/min, UV-254 nm, n-hexane/isopropyl alcohol 10 + 3 + 20% CH_2_Cl_2_, t_major_: 8.0 min, t_minor_: 7.3 min, 42% *ee*.

**3f** Yield 90%; m.p. 223-225 °C; ^1^H NMR (250 MHz, CDCl_3_) δ 0.80-1.80 (m, 10 H), 3.18 (dd, *J*_1_ = 3.4 Hz, *J*_2_ = 8.9 Hz, 1 H), 3.25 (d, *J* = 9.0 Hz, 1 H), 3.51-3.66 (m, 1 H), 4.63 (d, *J* = 3.3 Hz, 1 H), 5.09 (s, 1 H), 7.04-7.26 (m, 6 H); ^13^C NMR (63 MHz, CDCl_3_) δ 24.73, 25.54, 27.87, 45.22, 45.53, 50.57, 51.62, 81.42, 122.57, 123.82, 128.19, 128.39, 130.07, 130.19, 130.71, 131.01, 135.70, 137.08, 139.20, 141.82, 175.94, 177.25; IR (KBr) 3478 (m), 2941 (m), 2855 (m), 1767 (w), 1687 (s), 1572 (w), 1453 (s), 1397 (m), 1369 (s), 1348 (m), 1257 (m), 1187 (s), 1145 (m), 987 (w), 938 (m), 833 (w), 790 (m), 776 (m) cm^-1^; Anal. Calcd for C_24_H_21_Cl_2_NO_3_: C, 65.17; H, 4.79; N, 3.17; Found: C, 65.01; H, 4.91; N, 3.11. HPLC: CHIRALPAK IA, 250 x 4.6 mm (l x i.d.), flow-rate 0.7 mL/min, UV-254 nm, n-hexane/isopropyl alcohol 10 + 3 + 20% CH_2_Cl_2_, t_major_: 7.8 min, t_minor_: 11.0 min, 19% *ee*.

**3g** Yield 95%; m.p. 204-207 °C (Lit. 211-213°C) [11]; ^1^H NMR (250 MHz, CDCl_3_) δ 3.13 (d, *J* = 8.8 Hz, 1 H), 3.33 (dd, *J*_1_ = 3.5 Hz, *J*_2_ = 8.8 Hz, 1 H), 4.27 (s, 2 H), 4.42 (s, 1 H), 4.71 (d, *J* = 3.3 Hz, 1 H), 6.69-6.73 (m, 2 H), 6.96-7.42 (m, 10 H), 7.65-7.68 (m, 1 H); ^13^C NMR (63 MHz, CDCl_3_) δ 42.25, 44.35, 47.49, 50.56, 77.00, 120.66, 120.77, 123.63, 124.38, 126.68, 126.77, 127.15, 127.29, 127.47, 127.85, 128.44, 134.49, 136.35, 139.25, 140.65, 142.67, 176.02, 177.52; IR (KBr) 3362 (m), 3036 (w), 2962 (w), 1768 (w), 1684 (s), 1458 (m), 1432 (m), 1399 (m), 1352 (w), 1340 (w), 1316 (w), 1251 (m), 1181 (m), 1146 (w), 1077 (w), 985 (w), 936 (w), 905 (w), 876 (w), 826 (w), 768 (m), 749 (m), 702 (m) cm^-1^; Anal. Calcd for C_25_H_19_NO_3_: C, 78.72; H, 5.02; N, 3.67; Found: C, 78.50; H, 5.11; N, 3.48. HPLC: CHIRALPAK IA, 250 x 4.6 mm (l x i.d.), flow-rate 0.7 mL/min, UV-254 nm, n-hexane/isopropyl alcohol 10 + 3 + 20% CH_2_Cl_2_, t_major_: 9.6 min, t_minor_: 9.0 min, 20% *ee*.

**3h** Yield 85%; m.p. 220-221 °C; ^1^H NMR (250 MHz, CDCl_3_) δ 3.14 (d, *J* = 9.3 Hz, 1 H), 3.35 (dd, *J*_1_ = 3.4 Hz, *J*_2_ = 9.1 Hz, 1 H), 4.48 (s, 1 H), 4.77 (d, *J* = 3.5 Hz, 1 H), 6.18 (s, 1 H), 6.59-6.64 (m, 2 H), 6.82-6.87 (m, 2 H), 7.15-7.39 (m, 12 H), 7.53-7.56 (m, 1 H), 7.66-7.69 (m, 1 H); ^13^C NMR (63 MHz, CDCl_3_) δ 44.30, 47.31, 50.20, 58.47, 77.06, 120.66, 121.31, 123.57, 124.80, 126.65, 126.75, 127.29, 127.42, 127.52, 127.76, 127.96, 128.25, 128.29, 128.49, 136.55, 136.79, 136.92, 139.42, 141.36, 142.88, 175.76, 177.44; IR (KBr) 3516 (w), 3068 (w), 1772 (w), 1700 (s), 1497 (w), 1458 (m), 1390 (w), 1353 (m), 1253 (w), 1193 (w), 1173 (m), 772 (m), 756 (m), 702 (m) cm^-1^; Anal. Calcd for C_31_H_23_NO_3_: C, 81.38; H, 5.07; N, 3.06; Found: C, 81.25; H, 5.27; N, 2.90. HPLC: CHIRALPAK IA, 250 x 4.6 mm (l x i.d.), flow-rate 0.7 mL/min, UV-254 nm, n-hexane/isopropyl alcohol 10 + 3 + 20% CH_2_Cl_2_, t_major_: 11.3 min, t_minor_: 10.8 min, 26% *ee.*

**3i** Yield 70%; m.p. 215-217 °C; ^1^H NMR (250 MHz, CDCl_3_) δ 3.27 (d, *J* = 8.5 Hz, 1 H), 3.48 (dd, *J*_1_ = 3.5 Hz, *J*_2_ = 8.5 Hz, 1 H), 4.52 (s, 1 H), 4.83 (d, *J* = 3.5 Hz, 1 H), 6.36-6.42 (m, 2 H), 7.20-7.35 (m, 5 H), 7.40-7.46 (m, 3 H), 7.54-7.57 (m, 1 H), 7.72-7.76 (m, 1 H); ^13^C NMR (63 MHz, CDCl_3_) δ 44.80, 47.67, 50.82, 77.30, 120.93, 121.07, 123.01, 123.79, 124.70, 126.92, 127.00, 127.28, 127.39, 127.79, 129.79, 132.36, 136.55, 138.68, 140.87, 142.10, 175.28, 176.77; IR (KBr) 3410 (w), 3043 (w), 2960 (w), 1775 (w), 1703 (s), 1489 (s), 1458 (m), 1389 (m), 1298 (w), 1261 (w), 1244 (w), 1173 (s), 1136 (w), 1070 (w), 1012 (w), 920 (w), 907 (w), 876 (w), 825 (w), 770 (m), 728 (m) cm^-1^; Anal. Calcd for C_24_H_16_BrNO_3_: C, 64.59; H, 3.61; N, 3.14; Found: C, 64.34; H, 3.79; N, 2.91. HPLC: CHIRALPAK IA, 250 x 4.6 mm (l x i.d.), flow-rate 0.7 mL/min, UV-254 nm, n-hexane/isopropyl alcohol 10 + 3 + 20% CH_2_Cl_2_, t_major_: 13.7 min, t_minor_: 14.3 min, 13% *ee*.

**3j** Yield 82%; m.p. 215-217 °C; ^1^H NMR (250 MHz, CDCl_3_) δ 3.25 (d, *J* = 8.8 Hz, 1 H), 3.47 (dd, *J*_1_ = 3.5 Hz, *J*_2_ = 8.8 Hz, 1 H), 3.75 (s, 3 H), 4.55 (s, 1 H), 4.83 (d, *J* = 3.5 Hz, 1 H), 6.35-6.41 (m, 2 H), 6.77-6.84 (m, 2 H), 7.19-7.35 (m, 5 H), 7.39-7.43 (m, 1 H), 7.54-7.57 (m, 1 H), 7.71-7.75 (m, 1 H); ^13^C NMR (63 MHz, CDCl_3_) δ 44.82, 47.60, 50.73, 55.42, 77.31, 114.49, 120.89, 121.12, 123.40, 123.77, 124.71, 126.85, 126.94, 127.22, 127.33, 127.47, 136.68, 138.85, 140.99, 142.27, 159.78, 175.92, 177.43; IR (KBr) 3398 (w), 3071 (w), 2968 (w), 1776 (w), 1695 (s), 1610 (w), 1513 (s), 1458 (m), 1446 (m), 1405 (m), 1364 (w), 1307 (m), 1258 (s), 1195 (s), 1176 (s), 1165 (s), 1136 (m), 1104 (w), 1072 (w), 1024 (m), 989 (w), 961 (w), 919 (m), 879 (w), 827 (m), 770 (s), 752 (m), 744 (m), 731 (m), 707 (w) cm^-1^; Anal. Calcd for C_25_H_19_NO_4_: C, 75.55; H, 4.82; N, 3.52; Found: C, 75.29; H, 5.04; N, 3.32. HPLC: CHIRALPAK IA, 250 x 4.6 mm (l x i.d.), flow-rate 0.7 mL/min, UV-254 nm, n-hexane/isopropyl alcohol 10 + 3 + 20% CH_2_Cl_2_, t_major_: 12.6 min, t_minor_: 13.1 min, 32% *ee*.

**3k** Yield 13 and 65% respectively; m.p. 226-228 °C; ^1^H NMR (250 MHz, CDCl_3_) δ 0.51 (sp, *J* = 6.7 Hz, 1 H), 0.75 (d, *J* = 6.5 Hz, 3 H), 0.76 (d, *J* = 6.5 Hz, 3 H), 1.08 (d, *J* = 7.0 Hz, 3 H), 1.08 (d, *J* = 7.0 Hz, 3 H), 2.41 (sp, *J* = 6.7 Hz, 1 H), 3.44 (d, *J* = 9.5 Hz, 1 H), 3.62 (dd, *J*_1_ = 3.5 Hz, *J*_2_ = 9.6 Hz, 1 H), 4.48 (s, 1 H), 4.85 (d, *J* = 3.5 Hz, 1 H), 7.06-7.40 (m, 9 H), 7.59-7.62 (m, 1 H), 7.68-7.71 (m, 1 H); ^13^C NMR (63 MHz, CDCl_3_) δ 23.70, 23.80, 24.78, 24.82, 27.51, 29.42, 44.16, 48.06, 50.95, 77.00, 120.68, 121.26, 123.58, 123.66, 124.32, 124.78, 126.38, 126.68, 126.78, 127.39, 127.47, 130.17, 137.34, 139.57, 141.63, 143.04, 144.84, 146.82, 175.90, 177.48; IR (KBr) 3526 (m), 3071 (w), 3032 (w), 2966 (s), 2926 (m), 2868 (m), 1775 (m), 1701 (s), 1596 (w), 1459 (s), 1380 (s), 1299 (w), 1262 (w), 1243 (w), 1168 (s), 1077 (w), 1055 (w), 1025 (w), 997 (w), 940 (w), 918 (m), 878 (w), 806 (m), 770 (m), 756 (m), 732 (m) cm^-1^; Anal. Calcd for C_30_H_29_NO_3_: C, 79.80; H, 6.47; N, 3.10; Found: C, 79.60; H, 6.58; N, 2.94. HPLC: CHIRALPAK IA, 250 x 4.6 mm (l x i.d.), flow-rate 0.7 mL/min, UV-254 nm, n-hexane/isopropyl alcohol 10 + 0.25 + 5% CH_2_Cl_2_, t_major_: 24.1 min, t_minor_: 28.5 min, 76 and 51% *ee* respectively.

**3l** Yield 77%; m.p. 250-252 °C; ^1^H NMR (250 MHz, CDCl_3_) δ 0.65 (sp, *J* = 6.6 Hz, 1 H), 0.77 (d, *J* = 6.5 Hz, 3 H), 0.85 (d, *J* = 6.5 Hz, 3 H), 1.07 (d, *J* = 6.8 Hz, 3 H), 1.09 (d, *J* = 6.8 Hz, 3 H), 2.42 (sp, *J* = 6.9 Hz, 1 H), 3.57 (dd, *J*_1_ = 3.4 Hz, *J*_2_ = 9.6 Hz, 1 H), 3.67 (d, *J* = 9.5 Hz, 1 H), 4.78 (d, *J* = 3.5 Hz, 1 H), 5.01 (s, 1 H), 7.07-7.36 (m, 9 H); ^13^C NMR (63 MHz, CDCl_3_) δ 23.64, 23.84, 24.65, 24.73, 27.92, 29.48, 45.05, 46.20, 51.53, 81.29, 122.56, 123.79, 123.93, 124.31, 126.28, 128.27, 128.77, 130.08, 130.29, 130.55, 131.04, 131.21, 136.13, 137.55, 139.99, 142.24, 144.86, 146.55, 175.28, 176.45; IR (KBr) 3503 (m), 2967 (s), 2928 (w), 2867 (w), 1777 (m), 1702 (s), 1594 (w), 1572 (m), 1452 (s), 1424 (w), 1372 (s), 1279 (w), 1257 (m), 1186 (s), 1094 (w), 1056 (w), 996 (w), 966 (w), 936 (m), 920 (w), 902 (w), 878 (w), 828 (w), 808 (m), 788 (w), 772 (m), 746 (m), 715 (w) cm^-1^; Anal. Calcd for C_30_H_27_Cl_2_NO_3_: C, 69.23; H, 5.23; N, 2.69; Found: C, 69.18; H, 5.37; N, 2.53. HPLC: CHIRALPAK IA, 250 x 4.6 mm (l x i.d.), flow-rate 0.7 mL/min, UV-254 nm, n-hexane/isopropyl alcohol 10 + 1 + 5% CH_2_Cl_2_, t_major_: 10.1 min, t_minor_: 12.1 min, 34% *ee*.

**3m** Yield 76%; m.p. 241-243 °C; ^1^H NMR (250 MHz, CDCl_3_) δ 0.61 (sp, *J* = 6.4 Hz, 1 H), 0.78 (d, *J* = 6.5 Hz, 3 H), 0.84 (d, *J* = 6.8 Hz, 3 H), 1.09 (d, *J* = 7.0 Hz, 6 H), 2.41 (sp, *J* = 6.9 Hz, 1 H), 3.44 (d, *J* = 9.5 Hz, 1 H), 3.67 (dd, *J*_1_ = 3.4 Hz, *J*_2_ = 9.4 Hz, 1 H), 4.59 (br s, 1 H), 5.95 (d, *J* = 3.3 Hz, 1 H), 7.09-7.41 (m, 7 H), 7.51-7.55 (m, 1 H), 7.59-7.63 (m, 1 H); ^13^C NMR (63 MHz, CDCl_3_) δ 23.74, 24.70, 27.91, 29.47, 37.17, 46.56, 50.06, 77.16, 119.40, 120.04, 123.77, 124.33, 126.21, 127.44, 128.03, 128.27, 128.40, 129.95, 130.31, 131.32, 134.50, 136.29, 143.99, 144.87, 145.17, 146.60, 174.66, 177.05; IR (KBr) 3504 (m), 2966 (m), 2929 (m), 2869 (w), 1774 (w), 1703 (s), 1578 (w), 1456 (s), 1424 (w), 1365 (s), 1278 (w), 1243 (m), 1191 (s), 1111 (w), 1056 (w), 969 (w), 937 (w), 897 (w), 876 (w), 828 (w), 800 (m), 781 (m), 749 (w), 734 (w) cm^-1^; Anal. Calcd for C_30_H_27_Cl_2_NO_3_: C, 69.23; H, 5.23; N, 2.69; Found: C, 68.96; H, 5.22; N, 2.43. HPLC: CHIRALPAK IA, 250 x 4.6 mm (l x i.d.), flow-rate 0.7 mL/min, UV-254 nm, n-hexane/isopropyl alcohol 10 + 1 + 5% CH_2_Cl_2_, t_major_: 8.5 min, t_minor_: 11.4 min, 54% *ee* (96% *ee* after recrystallization).

**General procedure for the kinetics study of the reaction between *N*-phenylmaleimide (2a) and anthrone (1a).** All ^1^H NMR were recorded on a 250 MHz spectrometer. The reactions were carried out in a 5 mm standard NMR tube containing 0.1 mmol *N*-phenylmaleimide (**2a**) (17.3 mg), 0.11 mmol anthrone (**1a**) (1.1 eq, 21.4 mg) and 1 mol % of **8a** (0.001 mmol, 0.46 mg), 5 mol % of **8a**•H^+^•TFPB^-^ (0.005 mmol, 6.6 mg) or without catalyst in 0.7 mL deuterated dichloromethane at room temperature. The percentages of conversion were determined by integrating selected resonances for the product and unreacted *N*-phenylmaleimide (**2a**): **3a**, δ_H_ (m, 2H) = 6.43-6.51 ppm; *N*-phenylmaleimide (**2a**), δ_H_ (s, 2H) = 6.84 ppm. All reactions were repeated and the results were averaged.

**Table 5:** Optimization of the Diels-Alder reaction with catalyst **8a**.

| entry^a^ | catalyst amount [mol %] | solvent | Temperature [°C] | Reaction time [h] | yield [%]^b^ | ee [%]^c^ |
| --- | --- | --- | --- | --- | --- | --- |
| 1 | 25 | CH_2_Cl_2_ | 23 | 1 | 90 | 11 |
| 2 | 25 | CH_2_Cl_2_ | -25 | 24 | 87 | 17 |
| 3 | 25 | toluene | -25 | 24 | 87 | 15 |
| 4 | 25 | CH_2_Cl_2_ | -60 | 66 | 78 | 33 |
| 5 | 25 | CHCl_3_ | -60 | 66 | 60 | 24 |
| **6** | **25** | **CH_2_Cl_2_** | **-70** | **96** | **83** | **35** |
| 7 | 25 | CH_2_Cl_2_ | -70 | 120 | 80 | 35 |
| 8 | 20 | CH_2_Cl_2_ | -70 | 96 | 56 | 34 |
| 9 | 25 | toluene | -70 | 96 | <30 | n.d. |
| 10 | 25 | CH_2_Cl_2_ | -80 | 96 | 24 | 10 |

^a^All reactions were carried out using 0.1 mmol maleimide **2a**, 1.1 equiv anthrone (**1a**) and catalyst **8a** in 1 mL abs. solvent. ^b^Isolated yield after column chromatography. ^c^Enantiomeric excess was determined by HPLC using Chiralpak IA column.

Spectroscopic Data of Compound **9b**

Spectroscopic Data of Compound **9c**

Spectroscopic Data of Compound **8b***HCl

Spectroscopic Data of Compound **8b**

Spectroscopic Data of Compound **8c***HCl

Spectroscopic Data of Compound **8c**

Spectroscopic Data of Compound *ent*-**8d***HCl

Spectroscopic Data of Compound *ent*-**8d**

Spectroscopic Data of Compound **1b**

Spectroscopic Data of Compound **1c**

Spectroscopic Data of Compound **2b**

GC-MS of Compound **2b**

Spectroscopic Data of Compound **2c**

Spectroscopic Data of Compound **2f**

Spectroscopic Data of Compound **2g**

Spectroscopic Data of Compound **2h**

Spectroscopic Data of Compound **2i**

Spectroscopic Data of Compound **3a**

Spectroscopic Data of Compound **3b**

Spectroscopic Data of Compound **3c**

Spectroscopic Data of Compound **3d**

Spectroscopic Data of Compound **3e**

Spectroscopic Data of Compound **3f**

Spectroscopic Data of Compound **3g**

Spectroscopic Data of Compound **3h**

Spectroscopic Data of Compound **3i**

Spectroscopic Data of Compound **3j**

Spectroscopic Data of Compound **3k**

Spectroscopic Data of Compound **3l**

Spectroscopic Data of Compound **3m**

Chromatogramm on chiral column of **3a**

*Racemic*

*Catalyzed*

Chromatogramm on chiral column of **3b**

*Racemic*

*Catalyzed*

Chromatogramm on chiral column of **3c**

*Racemic*

*Catalyzed*

Chromatogramm on chiral column of **3d**

*Racemic*

*Catalyzed*

Chromatogramm on chiral column of **3e**

*Racemic*

*Catalyzed*

Chromatogramm on chiral column of **3f**

*Racemic*

*Catalyzed*

Chromatogramm on chiral column of **3g**

*Racemic*

*Catalyzed*

Chromatogramm on chiral column of **3h**

*Racemic*

*Catalyzed*

Chromatogramm on chiral column of **3i**

*Racemic*

*Catalyzed*

Chromatogramm on chiral column of **3j**

*Racemic*

*Catalyzed*

Chromatogramm on chiral column of **3k**

*Racemic*

*Catalyzed at room temperature*

*Catalyzed at -70 °C*

Chromatogramm on chiral column of **3l**

*Racemic*

*Catalyzed*

Chromatogramm on chiral column of **3m**

*Racemic*

*Catalyzed*

*Recrystallized*

**X-Ray Data of compound 3k**

Empirical formula C_30_H_29_NO_3_

Formula weight 451.54

Temperature 163(2) K

Wavelength 0.71073 Å

Crystal system Orthorhombic

Space group *P* 2_1_2_1_2_1_

Unit cell dimensions a = 11.2325(18) Å alpha = 90°.

b = 13.594(3) Å beta = 90°.

c = 15.866(4) Å gamma = 90°.

Volume 2422.6(9) Å^3^

Z 4

Density (calculated) 1.238 Mg/m^3^

Absorption coefficient 0.079 mm^-1^

F(000) 960

Crystal size 0.40 x 0.20 x 0.17 mm

Theta range for data collection 1.97 to 27.45°

Limiting indices -14<=h<=14, -26<=k<=17, -19<=l<=19

Reflections collected / unique 29990 / 2944 [R(int) = 0.1612]

Completeness to theta = 27.45 94.3%

Absorption correction None

Refinement method Full-matrix least-squares on F^2^

Data / restraints / parameters 2944 / 0 / 311

Goodness-of-fit on F^2^ 1.022

Final R indices [I>2sigma(I)] R1 = 0.0542, wR2 = 0.0955

R indices (all data) R1 = 0.1409, wR2 = 0.1177

Largest diff. peak and hole 0.171 and -0.191 e.Å^-3^

**X-Ray Data of compound 3m**

Empirical formula C_30_H_27_Cl_2_NO_3_

Formula weight 520.43

Temperature 168(2) K

Wavelength 0.71073 Å

Crystal system Triginal

Space group *P* 3_2_

Unit cell dimensions a = 21.483(3) Å alpha = 90°.

b = 21.483(3) Å beta = 90°.

c = 14.924(3) Å gamma = 90°.

Volume 5964.8(15) Å^3^

Z 9

Density (calculated) 1.304 Mg/m^3^

Absorption coefficient 0.277 mm^-1^

F(000) 2448

Crystal size 0.9 x 0.11 x 0.10 mm

Theta range for data collection 1.09 to 28.29°

Limiting indices -28<=h<=28, -26<=k<=26, -19<=l<=19

Reflections collected / unique 88688 / 18073 [R(int) = 0.1751]

Completeness to theta = 28.29 94.0%

Absorption correction Empirical

Max. And min. transmission 1.000 and 0.888

Refinement method Full-matrix least-squares on F^2^

Data / restraints / parameters 18073 / 1 / 974

Goodness-of-fit on F^2^ 0.889

Final R indices [I>2sigma(I)] R1 = 0.0604, wR2 = 0.0948

R indices (all data) R1 = 0.1820, wR2 = 0.1194

Absolute structure parameter -0.03(5)

Largest diff. peak and hole 0.550 and -0.311 e.Å^-3^

**References**

1. Scheurer, A.; Mosset, P.; Saalfrank, R. W. *Tetrahedron: Asymmetry* **1999,** *10,* 3559–3570. doi:[10.1016/S0957-4166(99)00353-5](http://dx.doi.org/10.1016/S0957-4166(99)00353-5)
2. Akalay, D.; Dürner, G.; Bats, J. W.; Bolte, M.; Göbel, M. W. *J. Org. Chem.* **2007,** *72,* 5618–5624. doi:[10.1021/jo070534j](http://dx.doi.org/10.1021/jo070534j)
3. Prinz, H.; Wiegrebe, W.; Müller, K. *J. Org. Chem.* **1996,** *61,* 2853–2856. doi:[10.1021/jo9520351](http://dx.doi.org/10.1021/jo9520351)
4. House, H. O.; Hrabie, J. A.; VanDerveer, D. *J. Org. Chem.* **1986,** *51,* 921–929. doi:[10.1021/jo00356a031](http://dx.doi.org/10.1021/jo00356a031)
5. **2b**: Walker, M. A. *J. Org. Chem.* **1995,** *60,* 5352–5355. doi:[10.1021/jo00121a070](http://dx.doi.org/10.1021/jo00121a070)
6. **2c**, **2f**–**h**: Cava, M. P.; Deana, A. A.; Muth, K.; Mitchell, M. J. *Org. Synth.* **1961,** *41,* 93–95.
7. **2i**: Fielding, M. R.; Grigg, R.; Sridharan, V.; Thornton-Pett, M.; Urch, C. J. *Tetrahedron* **2001,** *57,* 7737–7748. doi:[10.1016/S0040-4020(01)00740-2](http://dx.doi.org/10.1016/S0040-4020(01)00740-2)
8. Betschmann, P.; Sahli, S.; Diederich, F.; Obst, U.; Gramlich, V. *Helv. Chim. Acta* **2002,** *85,* 1210–1245. doi:[10.1002/1522-2675(200205)85:5<1210::AID-HLCA1210>3.0.CO;2-T](http://dx.doi.org/10.1002/1522-2675(200205)85:5<1210::AID-HLCA1210>3.0.CO;2-T)
9. Fronborg, J.; Magnusson, G.; Thoren, S. *J. Org. Chem.* **1975,** *40,* 1595–1601. doi:[10.1021/jo00899a017](http://dx.doi.org/10.1021/jo00899a017)
10. Pyriadi, T. M. *J. Org. Chem.* **1972,** *37,* 4184–4186. doi:[10.1021/jo00798a049](http://dx.doi.org/10.1021/jo00798a049)
11. Uemae, K.; Masuda, S.; Yamamoto, Y. *J. Chem. Soc., Perkin Trans. 1* **2001,** 1002–1006. doi:[10.1039/b100961n](http://dx.doi.org/10.1039/b100961n)
